# An origami-based technique for simple, effective and inexpensive fabrication of highly aligned far-field electrospun fibers

**DOI:** 10.1038/s41598-023-34015-z

**Published:** 2023-05-01

**Authors:** Hamed Hosseinian, Martin Jimenez-Moreno, Mazhar Sher, Aida Rodriguez-Garcia, Sergio O. Martinez-Chapa, Samira Hosseini

**Affiliations:** 1 School of Engineering and Sciences, Tecnologico de Monterrey, 64849 Monterrey, NL Mexico; 2 Department of Agricultural and Biosystems Engineering, South Dakota State University, Brookings, SD 57007 USA; 3 Universidad Autónoma de Nuevo León, Facultad de Ciencias Biológicas, Instituto de Biotecnología, Ciudad Universitaria, San Nicolás de los Garza, 66455 San Nicolás, Nuevo Leon Mexico; 4 Writing Lab, Institute for the Future of Education, Tecnologico de Monterrey, 64849 Monterrey, NL Mexico

**Keywords:** Materials science, Biomaterials, Materials for devices, Materials chemistry, Biomaterials

## Abstract

Fabrication of highly aligned fibers by far-field electrospinning is a challenging task to accomplish. Multiple studies present advances in the alignment of electrospun fibers which involve modification of the conventional electrospinning setup with complex additions, multi-phased fabrication, and expensive components. This study presents a new collector design with an origami structure to produce highly-aligned far-field electrospun fibers. The origami collector mounts on the rotating drum and can be easily attached and removed for each round of fiber fabrication. This simple, effective, and inexpensive technique yields high-quality ultra-aligned fibers while the setup remains intact for other fabrication types. The electrospun poly(ɛ-caprolactone) (PCL) fibers were assessed by scanning electron microscope (SEM), fiber diameter distribution, water contact angle (WCA), Fast Fourier Transform analysis (FFT), surface plot profile, and pixel intensity plots. We thoroughly explored the impact of influential parameters, including polymer concentration, injection rate, collector rotation speed, distance from the collector to the tip, and needle gauge number on fibers’ quality and alignment. Moreover, we employed machine learning algorithms to predict the outcomes and classify the high-quality fibers instead of low-quality productions.

## Introduction

Electrospinning technique has been broadly hired to generate fibers from nano- to micro-scale from various polymers, co-polymers, and combinations of polymers. Commonly, far-field electrospinning setup is coupled with a rotating drum to create aligned fibers. However, creation of aligned fibers is a function of multiple elements to be controlled and not only depend on the rotating drums. High control over fibers’ orientation is crucial for widening the range of fiber applications, including drug delivery^1^, tissue engineering^2–4^, wound healing^5^, biosensors^6,7^, nerve regeneration^8,9^, and other biomedical application^10–12^. The conventional cylindrical mandrel, typically used for aligned fibers, comes with certain restrictions, including a lack of close control over the fiber deposition and alignment^13^. It is also reported that mandrel rotation may result in random fiber deposition at low speeds, while higher mandrel velocity could lead to well-oriented fibers. Nonetheless, a fine tuning of the collector’s speed is mandatory since high collector velocity worsen the fiber’s alignment due to a lack of control over the degree of anisotropy^14,15^. Moreover, producing highly aligned fibers may also require recruiting a complex setup (rotating the auxiliary electrodes around the needle axis)^16^, costly additions (adding a parallel electrode collector)^17^, and multiple stages of fabrication (post-drawing)^18^.

Researchers have proposed versatile methods to form aligned fibers^19–23^. Recently, Cui et al. fabricated an aligned PCL membrane with chitosan to control the release of encapsulated ciprofloxacin. In this work, coaxial electrospinning was used to produce random/aligned fibers for wound healing application, even though the drug-loaded membrane did not exhibit a high alignment of the fibers^1^. In other studies by Hu et al.^24^, and Xu et al.^25^ highly aligned fibers were successfully fabricated via a thin disc collector with a diameter of 280 mm and 200 mm, respectively. This method can offer an enhanced orientation of fibers, nevertheless, this type of collector comes with a limited accessible area for collecting aligned fibers. Kador et al. covered a plastic cup with aluminum foil and placed a copper wire at the center of the cup as the central pin while both connected to the same ground. The authors claimed to have successfully removed voids and beads from the generated fibers, while prior processing to the electrospinning was needed^26^. Some researchers utilized auxiliary electrode setups to reach a high degree of control over the orientation and deposition of the electrospun fibers on the collector area^27^. For instance, Zaho et al. used a parallel electrode collector instead of a regular rotating drum and placed a positively charged copper ring between the collector and needle. Highly aligned nanofibers were produced for a long spinning time; however, additions make the setup rather complex and costly^28^. In a more recent work by Tindell et al., an accurate spatial control over fiber orientation was achieved by using magnetically-assisted electrospinning. By having multiple magnet configurations installed in the setup, a variety of fiber gradients were produced including highly aligned fibers within the magnetic region and smoothly aligned fibers within the nonmagnetic region^29^. The highly aligned fibers, in this study, are entirely dependent on the magnet configuration. Moreover, other studies fabricated nanofibers via lateral spinnerets which deposit the fibers on the collector from opposite directions^30,31^. For instance, in 2019 Tian et al. employed a conjugate electrospinning setup to achieve aligned microfibers by improving the setup’s conditions. The fabricated fibers benefited from important features including tunable magnetism, electrically anisotropic conduction, and enhanced fluorescence^32^.

While significant milestones were marked on the path to yielding highly aligned fibers, these setups involve modification of the conventional setup which can pose a challenge in shared laboratories where different research projects are conducted with the same set of devices. A methodology that avoids alteration of the original fabrication set up and offers a cost-effective procedure for fabrication of the aligned fibers is not only practical but also highly desirable.

In this study, we report the design and implementation of a new collector with an origami structure that has produced highly-oriented electrospun fibers from poly(ɛ-caprolactone) (PCL). This easy-to-fabricate collector addresses some of the challenges faced by previous researchers while eliminating complex, costly, and multi-phased fabrication setups. The fabricated fibers were the result of a variation of the experimental parameters in 243 rounds of fabrication. The produced fibers were thoroughly analyzed through scanning electron microscope (SEM), fiber diameter distribution, water contact angle (WCA), fast fourier transform analysis (FFT), surface plot profile, and pixel intensity plots. We have selected conditions that yielded poor, medium-quality, and high-quality fibers from the total of experimental outcomes. We further analyzed the influential parameters that could be considered major key players in the fabrication of aligned fibers. Moreover, we employed advanced statistical analyses, exploratory data analyses, logistic Regression, and decision trees to enhance further our understanding of the parameters by which bead-free highly-aligned fibers can be fabricated. This paper brings the area of material science and data science in unity by incorporating machine learning algorithms in the decision-making for fiber fabrication.

## Background of the study

The ability to manipulate fiber alignment allows more intricate and efficient anisotropic fiber fabrication. Manufacturing electrospun fibers with controlled orientation can significantly enhance their properties and potential applications of the fibers, making it a highly demanding goal in various fields. Several electrospinning setups for fiber alignment were proposed including rotating mandrel electrospinning, gap electrospinning, magnetic electrospinning, auxiliary electrode electrospinning, centrifugal electrospinning, and post-modification.

Zhang et al. fabricated PCL-based electrospun fibers with controlled orientation using a rotating mandrel electrospinning. The authors varied the rotational speed of the apparatus to achieve different degrees of orientation, with speeds of 500, 1000, and 2000 rpm. The proposed fibers in this study were conductive composite with added carbon nanotubes (CNTs). The results showed that the PCL electrospun fibers produced at a rotational speed of 500 rpm had the highest degree of alignment along the rotational direction. However, the produced fibers were mono-layer without accumulating further layers^33^. While this study offers insights on the fabrication of aligned fibers hinting at lower-speed suitability, it is a fundamental study that does not generate applicable fibers for mass-manufacturing or for a potential application. Noteworthy, preserving the alignment when several fiber layers are mounted on top of one another is a grand challenge to tackle, which is not targeted in this work.

Hu et al.^24^ and Xu et al.^25^ successfully fabricated highly aligned fibers via thin disc collector with a diameter of 280 mm and 200 mm, respectively. This method can offer an enhanced orientation of fibers, nevertheless, this type of collector comes with a limit of accessible area for fiber collection. The study by Courtney et al. revealed the relationship between rotation speed and alignment. The authors found that a certain mandrel velocity, specifically 3.0 m/s, was necessary to produce aligned fibers. Fabricated fibers at a mandrel velocity of 13.8 m/s demonstrated a high stretch however lost partial orientation. This improved alignment also led to enhanced mechanical properties^34^. While the study presented improved alignment, the high control over the orientation of the fibers was yet to achieve.

The Xie et al. study introduced aligned electrospun fiber scaffolds, mimicking the collagen structure fibers at the point where tendons attach to bone. Gap electrospinning was used to produce bone tissue scaffolds by the fabrication of aligned fibers between the gap and randomly deposited fibers on the plate. The production of aligned fibers resulted in high tensile strength compared with randomly deposited fibers, representing the mechanical architecture of the tendon-to-bone site^35^. In another study by Jha et al., a 3D scaffold with aligned fibers was created from PCL by air gap electrospinning. This setup included a pair of vertical piers connected between − 4.0 and − 16.0 kV. The authors noted that this method could be used to direct axon growth through the regeneration of nerves, specifically via filling gaps among severely damaged nerves^36^. Kishan et al. developed a specialized wheel made with 3D printing and wire struts to fabricate a mat with aligned fibers. This mat was intended for grafts of the tendon-bone enthesis. To achieve fiber alignment, the team used air-gap electrospinning process and designed a wheel collector with parallel copper wires. The wheel collector rotated slowly during the fiber deposition process, and the rotation rate was synchronized with the speed of electrospinning to maintain a consistent mesh thickness. A biodegradable polyurethane (BPUR) with a gradient of mechanical properties ranging from BPUR 50 (50% hard segment) to BPUR 10 (10% hard segment) was used in the direction of alignment^37^. This technique has several limitations, one of which is the gap width which has restricted the area for fiber production.

Tindell et al. reported the use of magnetic field during electrospinning which enabled precise control over the alignment of fibers. By adjusting the configuration of the magnets, various fiber gradients could be achieved, including well-oriented electrospun fibers within the magnetic field area and a smooth transition to random alignment as the fibers moved away from the field. This technique, known as magnetically-assisted electrospinning, advanced fiber orientation control in sub-millimeter regime, mimicking the natural structural gradients found in many interface tissues^29^. Abiona et al. produced well-aligned nanofibers from poly (ethylene oxide) (PEO) by incorporating a magnetic field into the electric field during the electrospinning process. This was achieved by placing a cylindrical magnet within the electric field. The resulting nanofibers were collected on the top of the magnet. The modified setup consisted of a cylindrical magnet placed vertically in front of a grounded aluminum foil. The team then used silicon wafer substrates placed on top, sides, and front of the magnet to collect the nanofibers^38^. This setup is a pioneering method that combines multiple techniques for achieving high level of fiber alignment; nonetheless, quite complex in nature. In general, when using magnetic field-assisted electrospinning with two parallel magnets, the deposition area becomes somewhat limited, but this limitation does not apply to setups with one magnet as a direct collector or other collector types^29,38,39^.

Zhao et al. employed a charged copper ring as an auxiliary device to alleviate bending instability and enhance alignment during electrospinning. The parallel electrode method (PEM) was modified by adding a positively charged ring between the parallel electrode collector and the needle, resulting in improved distribution of diameter and increased alignment of the produced nanofibers. A considerable improvement in fiber orientation was achieved due to the insertion of auxiliary ring electrodes within the electrospinning setup, with above 70% alignment degree as compared to ~ 45% yielded by the traditional electrospinning setups. Additionally, the researchers found that the degree of alignment remained above 35% even after 60 min of spinning, while it was less than 5% with standard gap electrospinning^28^. In another study by Grasl et al., auxiliary electrodes were used to fabricate electrospun fibers through rotation and voltage alternation during the electrospinning process. The authors claimed that applying a 40 Hz voltage was optimal for fabricating aligned fibers between the electrodes^16^. Auxiliary electrodes require additional parts ranging from simple insertions to more complex pieces. This added complexity and equipment can make auxiliary electrodes less accessible.

According to a study by Erickson et al., a new collector of parallel wires with a distance of 1.27 cm yielded oriented fibers with an alignment degree of 75% at a rotation speed of 108 rpm. This is significantly higher than the traditional plate method, which typically only yields 20% alignment. The collector used in this method was a circular device with a 40.5 cm diameter attached to wooden columns. The spinneret was accompanied by a needle tip with a 10.7 cm distance from the collector and was positioned at the center of the collector. The collector also featured 102 grounded wire electrodes, which were separated by a distance of 1.27 cm^40^. In 2017, Wang et al. used a unique collector system to fabricate highly oriented and multi-layer patterned meshes. This setup involved conductive iron wire rings which were horizontally positioned around the spinneret. The fibers were fabricated at 70 rpm collector velocity. To vary the distance between the collector and the spinneret, circular peripheral collectors with variable diameters were employed and the distance was altered from 9 to 14 cm. The high degree of fiber orientation achieved in this study provided excellent control over the drug release loaded within the matrix of the fibers^17^. The use of parallel electrode collectors is known to be instrumental in the fabrication of well-oriented fibers. However, the ability to create more complex structures and scaffolds using this technique is restricted due to the lack of flexibility in adjusting the collector's parameters.

Recently, Hsu et al. presented a new copper plate stretching module linked to a motorized reel to adjust the velocity of the collector. This post-modification tool was used to create aligned arrays of fibers made of polystyrene (PS) and poly (methyl methacrylate) (PMMA) from randomly oriented meshes. The researchers found that the processed PS and PMMA fibers showed substantial improvements in alignment, uniformity of diameter distribution, and increased wettability, as well as in surface properties and shape of the fibers^18^. Brennan et al. developed an automated system that post-draws PCL fibers immediately after they are deposited. This device improves fibers’ orientation by gathering them among a gap between parallel tracks, comparable to the process of gap electrospinning. The system then stretches the fibers individually before the solvent is fully evaporated, which enhances the alignment of macromolecules and improves the anisotropy and mechanical properties of the fibers. The study showed that the fibers’ orientation was considerably increased (from 15 to 83%) as a result of the increased drawing ratio. Additionally, the fabricated fibers through this technique preserved extension up to 42% and displayed greater roughness when compared to fibers aligned using traditional post-drawing techniques, which involve annealing and stretching^41^.

Despite considerable efforts, post-drawing treatments introduce multiple challenges, including complex setups and equipment, a decrease in porosity, the merging of fibers, and variations in the alignment of fibers in space. Apart from costly setups and multi-phased fabrication, one fundamental problem is the resource-limitedness of such customized systems as they may not come in handy to other researchers in need of far-field electrospinning setup. It is therefore evident that there is a need for a simpler, inexpensive, and effective method for fabricating aligned far-field electrospun fibers that minimized the setup alternation, therefore, prepared for other types of fabrication. In this study, we attempt to address these challenges.

## Methodology

### Materials, chemicals and reagents

Polycaprolactone (PCL, MW = 80,000 g/mol), Tetrahydrofuran (THF), and N,N dimethylformamide (DMF) were purchased from the Sigma-Aldrich (St. Louis, MO, USA). The materials were used without additional modification. Non-Stick Aluminum Foil (Reynolds Wrap®, USA) and blunt-end syringes (McMaster-Carr, USA) were purchased separately.

### Electrospinning procedure

**Figure 1:**
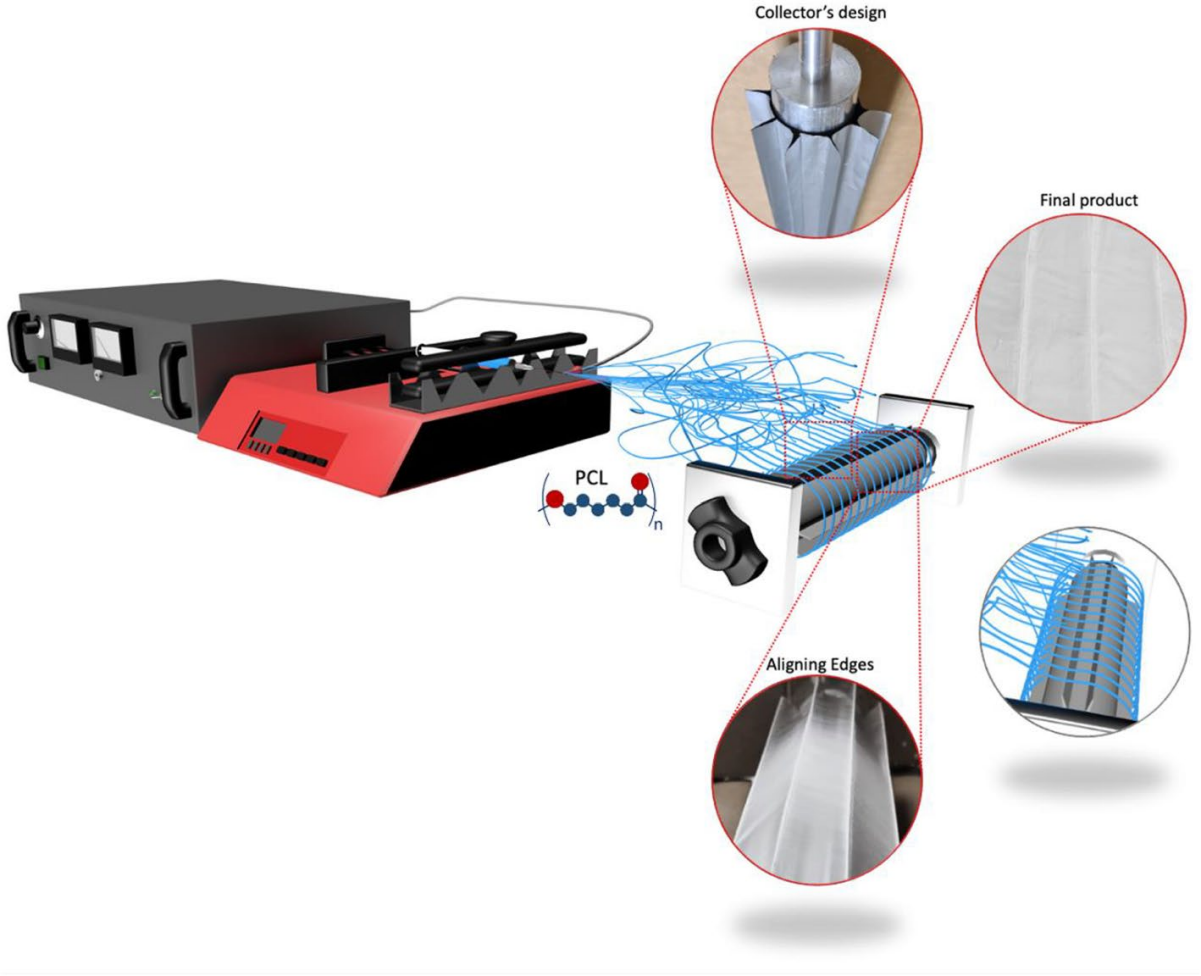
A representation of the far-field electrospinning setup equipped with an origami folded aluminum foil. The designed edges on the origami foils allowed controlled and high-quality alignment of the PCL fiber. Aligned fibers were fabricated without any extra addition to the original electrospinning setup.

Homogenous solutions of 5%, 10% and 15% wt% PCL at a volume ratio of 9:1 v/v (THF/DMF) were prepared at room temperature by continuously stirring the mixture for 2 h. A standard electrospinning setup containing a New Era Pump Systems, Inc (NY, USA) (Fig. 1), power supply with high voltage capacity (Spellman CZE1000R, USA), and metallic conductive collector (length = 28 cm, radius = 5 cm) were used to fabricate highly aligned PCL fibers.

High voltage power supply was featured in positive or negative polarities with outputs ranging from 1 to 30 kV, and the applied voltage was kept constant at 10 kV in all experiments. The humidity of the room was maintained at ~ 40% during the electrospinning process via Friedrich dehumidifier (D50BP, USA). A 5 ml syringe was used for each experiment to avoid cross-contamination. The syringe was made of compressed transparent plastic compatible to a cap with various needle gauges. The inner diameters of the stainless-steel dispensing needle were 0.026 in., 0.020 in., and 0.012 in. for needle gauge numbers 20, 22, and 25, respectively (Table 1). Throughout the electrospinning process, relative temperature and humidity were kept constant in the range of 28 ± 1 °C and 40–43%, respectively.

**Table 1:** Needles specifications.

Gauge	Needle type	Length	Inner diameter	Outer diameter	Angle
20	Blunt	1″	0.026″	0.036″	Straight
22	Blunt	1″	0.020″	0.028″	Straight
25	Blunt	1″	0.012″	0.020″	Straight

To find the influential factors in the alignment of fibers, we considered a range of parameters selected according to the latest related published papers. To plan the experimental phase of this work, we kept the needle type (blunt end), injection volume (5 ml), time (1.5 h), and voltage (10 kv) as constant, while variable factors were chosen to be polymer concentration (5%, 10% and 15%), the injection rate (0.3, 0.5 and 0.7 ml/h), the distance between the tip and the collector (10, 15 and 20 cm), collector rotation speed (8, 10, 12 rps), and needle gauge number (20, 22, and 25). The variable parameters were kept constant while other parameters have changed throughout iterations, marking 243 experiments to evaluate the pre-determined parameters precisely. The table below represents the combination of variable factors for the fabrication of better-aligned fibers (Table 2).

**Table 2:** Various parameters were considered, as shown below, to find the optimum alignment.

Constant parameters	Variable parameters	Range	Unit
Needle type (blunt end)	Polymer concentration	5%, 10%, 15%	wt%
Volume of injected solution (5 ml)	Injection rate	0.3, 0.5, 0.7	ml/h
Time (1.5 h)	Distance to the collector	10, 15, 20	Cm
Voltage (10 kV)	Collector rotation speed	8, 10, 12	RPS
Electrospinning setup	Needle gauge number	20, 22, 25	
Total experiments	243		

### Design and development of a rotating drum platform

The current study aimed at introducing a novel type of collector that yields highly aligned fibers that surpass the quality of the existing report, apart from ease of use and cost-effectivity. For that reason, we relied upon a regular rotating drum collector and mounted an origami folded design made of aluminum foil to secure better control over fiber deposition. This platform has prevented extra expensive additions and complex setups and was easy to dismount at any given point so that the setup can be used for other purposes. We present various angles of this design in Fig. 1 and Supplementary Fig. 1 for further clarity. The thickness of the aluminum foil was 0.6 mm. Upon folding in the desirable shape, it was mounted on the rotating drum and fixed by a scotch tape. The non-stick nature of the aluminum foil allowed the fibers to be effortlessly detached from the platform hence reusability of the origami-based collector. Before mounting the origami design on the drum, meshed fibers were produced using the rotating drum. For the fabrication of the highly aligned fibers, we have analyzed the width, and the height of the edges (1 cm and 1.5 cm, respectively) and, through several trials, selected the optimum design as presented in Supplementary Fig. 1. The fins’ height and width were optimized through multiple variation of the diameters whereby the alignment of the fibers was ensured and the space between the fins were sufficient for detachment of the fibers from the collector.

### Characterization of the meshed and aligned fibers

PCL fibers were imaged under high vacuum pressure using a scanning electron microscope (SEM) (EVOMA25, Germany) at a voltage of 15 kV in magnifications of 3.00 KX, 1.00 KX, 500 X, and 100 X. Formerly, the samples were gold sputter-coated by Quorum (Q150RES, England). The thickness of the coated layer was approximately 5 nm. Subsequently, PCL fibers were mounted on SEM sample holder with double side carbon tape. A total of 1200 individual SEM images were collected from the samples for more detailed analysis.

The diameter of the fibers was calculated using Image J software. For each sample, 40 measurements were performed (10 measurements for each SEM image and four magnifications) to plot the histogram. Additionally, the orientation of fibers was determined by Fiji (an extension of ImageJ). SEM images were cropped to a square shape for this analysis and processed in ImageJ software.

The water contact angle on the surface of the fibers was measured by DataPhysics instrument (OCA15EC, Germany) to calculate the wettability of the PCL electrospun fibers. Angles of PCL electrospun fiber samples were the results of 5 measurements for 3 water droplets deposited on the center and corners of individual samples.

The Fast Fourier Transform (FFT) analysis, surface plot profile, and pixel intensity plots were generated using Image J software. The directionality of the respective fibers was plotted using Fiji. Moreover, the topographic views of the fibers, which, in turn, indicate the alignment, were obtained from Image J software.

### Leveraging machine learning to classify fibers based on quality

**Scheme 1:**
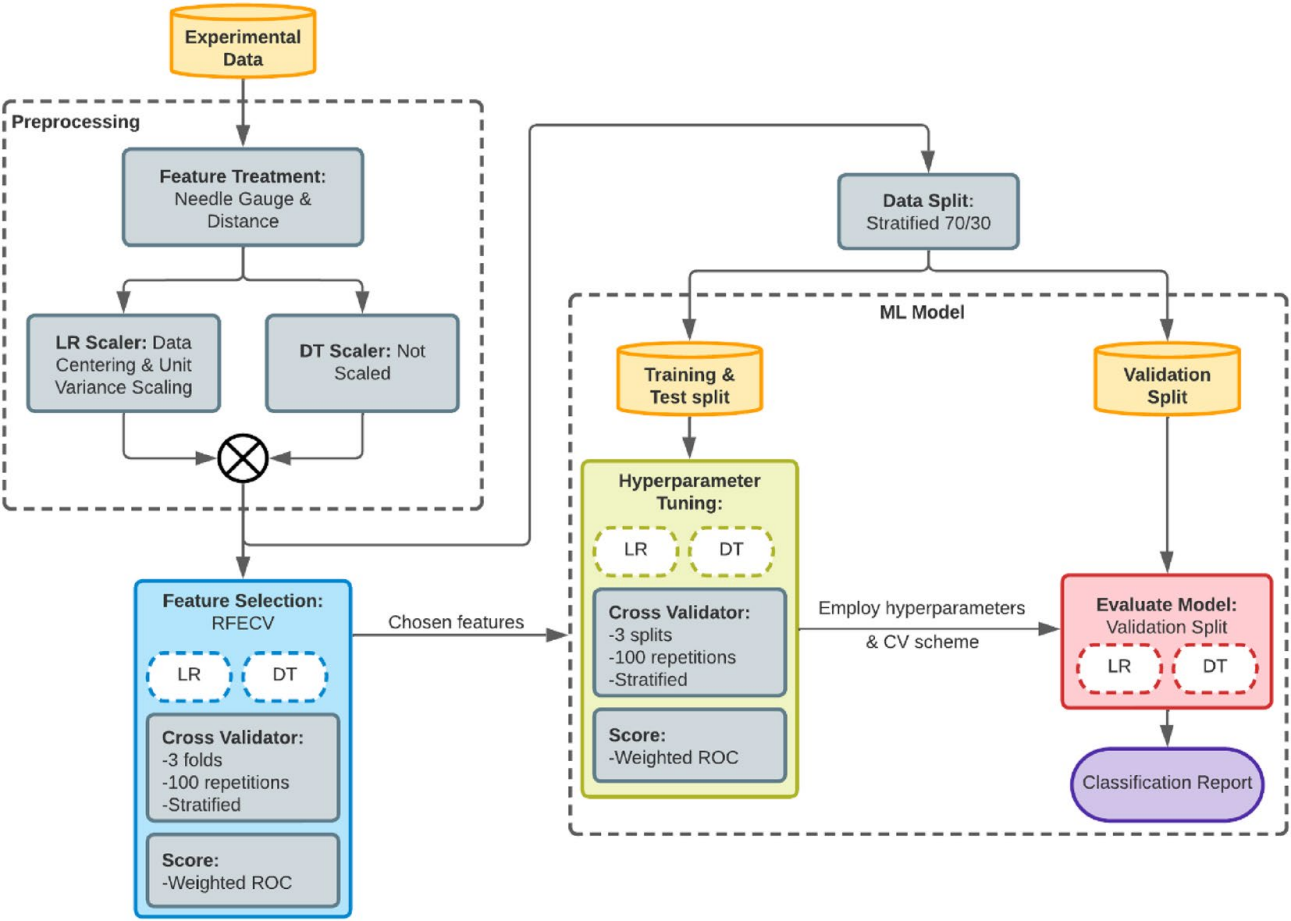
Machine learning pipeline for the analysis of the fibers.

We employ machine learning (ML) techniques to build up models that establish experimental parameter relationships with high-quality fiber production, which is briefly condensed in Scheme 1. From experimental data, fibers are classified as high quality (HQ)—or conversely as low quality (LQ)—when all the conditions are met according to the following criteria:

- If fibers display a high degree of orientation.
- If fibers demonstrate to be free from bead formation.
- If the fibers are homogenously deposited on the collector.
- If the fibers are homogenous in thickness.

Once the dataset is generated, we calculate Pearson correlation to find linear relations of the target label with the data and generate pair plot histograms using the Seaborn 0.12.2 library^42^. For the current study, we employ Logistic Regression (LR) and Decision Trees (DT) as glass box models that describe the relationship between experimental parameters and the attainability of good quality fibers. We employ open-sourced library Scikit-learn 1.2.0^43^ as tool for classification data analysis and Bokeh 3.0.3 as data visualization library^42^.

To ensure the predictive accuracy of ML models, features were treated the following way: rather than using needle gauge as a model feature; we employ its equivalent inner needle area value as an informative physical property, where gauges 20, 22, and 25 are taken respectively as 0.6, 0.41 and 0.26 mm^2^. Since it is expected that electric field intensity scales inversely with the length between needle tip and drum, we employ the reciprocal of the said distance as a general model feature. All features were standardized (with zero-mean and unit-standard deviation) when modeling with logistic regression and not scaled in the case of decision trees.

Feature selection was performed by recursive feature elimination with cross-validation (RFECV) to find the most important subset of features that provides the best generalization. We employ both LR and DT models as estimators, with the area under the receiver operating characteristic curve (ROC AUC) as prediction score and performing a hundred runs of stratified threefolds of cross validation runs. For machine learning modeling, 70% of the dataset is split for hyperparameter tuning, and 30% is reserved as unseen data for validation.

For the hyperparameter tuning stage, we employed the same cross-validation setup from feature selection to ensure accuracy and reduce the adverse effects of splitting a limited dataset. With ROC AUC as the target scorer, the C hyperparameter was tuned for the LR model, while leaf parameters (maximum tree depth, node’s minimum samples to split, minimum number of samples at leaf node) were tuned for DT case. Post-pruning was performed on the most representative tree by a minimal cost complexity algorithm to reduce non-critical classification instances^44^. The concluding models were evaluated according to common classification report metrics when predicting fiber quality with the validation set, and their results were compared to a random dummy classifier as the baseline. Accuracy, precision, recall and F1 metrics were defined according to classification outcomes, TP (True Positive), TN (True Negative), FP (False Positive) and FN (False Negative) by the following expressions:

$$Accuracy = \frac{TP + TN}{{TP + TN + FP + FN}}$$

$$Precision = \frac{TP}{{TP + FP}}$$

$$Recall = \frac{TP}{{TP + FN}}$$

$$F1 = 2\frac{Precision \times Recall}{{Precision + Recall}}$$

## Results and discussion

### Characterization of the aligned fibers by scanning electron microscopy (SEM)

**Figure 2:**
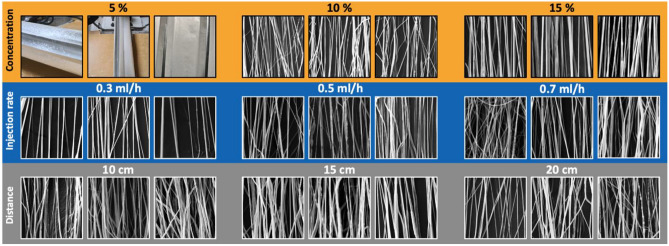
The main influential parameters in aligned fiber fabrication: Polymer concentration (5%, 10% and 15%), the injection rate (0.3, 0.5, and 0.7 ml/h), and the distance between the tip and the collector (10, 15, and 20 cm). Note, the images are selected SEM representations of the fibers. For complete comparison, see Supplementary Table 1.

As one of the main objectives of this study, we aimed to produce highly aligned bead-free fibers with a cost-effective and simple platform. The consistency and strength of the fibers were important qualities to ensure. As mentioned in the methodology section, several experimental parameters were kept constant while the rest were systematically changed to find the fine-tuning for fiber fabrication. Figure 2 represents the SEM results of the fiber fabrication in different polymer concentrations, injection rates, and distances to the collector. As can be seen, the 5% polymer solution resulted in poor quality production with a powder-like outcome rather than fibers. After repeating multiple experiments with this specific polymer concentration, we disqualified 5% as a suitable choice for fiber fabrication and continued with 10% and 15% polymer concentrations. Overall, we found the polymer concentration to play a crucial role in aligned fiber fabrication. The higher the polymer concentration, the higher the quality of the fibers was. Meanwhile, the SEM images show a more condensed fiber deposition as the injection rate increases, but the distance from the needle to the collector does not present any visual differences among the fibers.

### Fiber diameter range analysis of the aligned fibers

**Figure 3:**
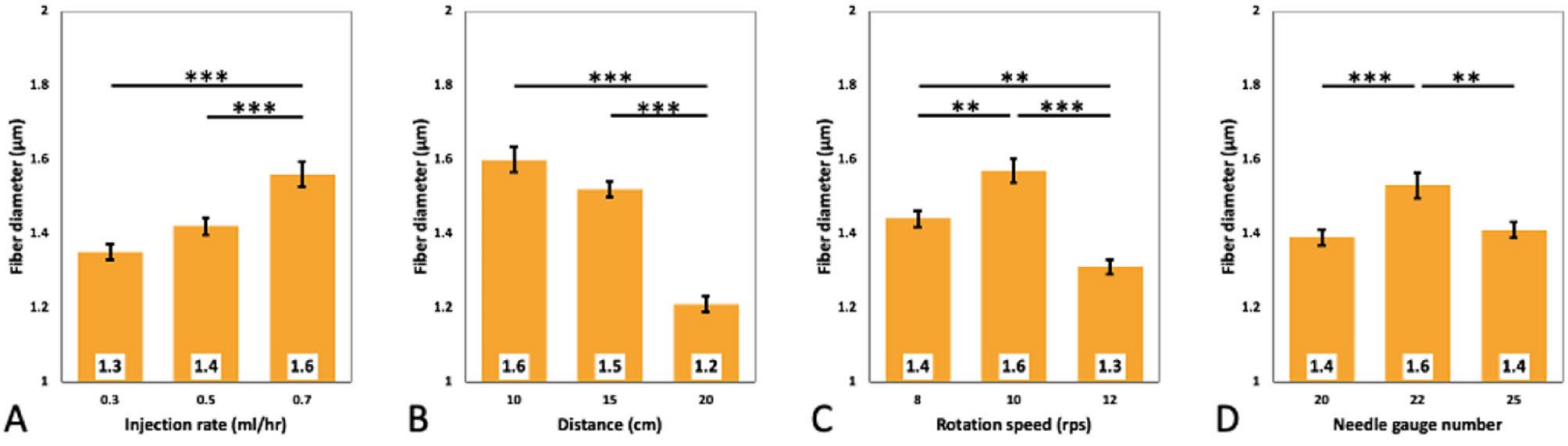
Fiber diameter distribution based on various influential electrospinning parameters. Bar graphs represent the effects of (**A**) Injection rate (ml/h), (**B**) needle-collector distance (cm), (**C**) drum rotation speed (rps), (**D**) needle gauge number on the fibers diameter. (Statistical significance was determined using one-way ANOVA with Tukey post-hoc analyses. ** and *** indicate the significance level of p < 0.01 and p < 0.001, respectively. Data is shown in mean ± standard error of the mean (SEM) (n = 1080).

Ongoing studies have presented a range of fiber sizes from micro- to nano-scale demanded by different biomedical applications^45–47^. Figure 3 represents the influence of applied parameters on the diameter range of the fabricated aligned PCL fibers. According to the results, PCL fiber diameter varied from 1.2 to 1.6 μm in general, which is in the range of recently reported measurements^48^. The average fiber diameter in total seems to be 1.4 μm, which is no different from the minimum and maximum we observed. The fibers seem to be highly reproducible as no major difference was seen in the fiber diameter range, and the same type of consistency was also observed in the SEM analyses (Fig. 1).

Our observations suggest that the PCL fiber diameter has continuously increased from 1.3 to 1.4 μm and 1.6 μm as the injection rate increased from 0.3 to 0.5 ml/h and 0.7 ml/h, respectively (Fig. 3A), which is an expected outcome considering the volume of the polymer solution ejected from the needle. Moreover, the distance to the collector seems to have an inverse impact on fiber diameter as the fibers systematically reduced in size as this distance became longer (10 cm, 15 cm, and 20 cm). Particularly, the 20 cm distance from the collector has yielded in significantly smaller fiber diameter (1.3 μm) compared to the rest (Fig. 3B). Statistical analysis of the measurements showed a significant difference in fiber diameter between 20 cm (distance between the needle and the collector) compared to 10 cm and 15 cm (p < 0.001). No statistical difference was observed when the needle-collector distance was set at 10 cm and 15 cm. This is in line with the findings of the literature as, for instance, Ghobeira et al. also reported thinner PCL fibers for longer distances between the needle tip and the collector^48^.

Another controlled parameter was the rotation speed of the drum collector, which seemed to be independent of the fiber diameter. The fiber diameters of 1.4 μm, 1.6 μm, and 1.3 μm were obtained from the drum’s rotation speed of 8 rps, 10 rps, and 12 rps, respectively (Fig. 3C). This finding, however, does not seem to agree with that of some of the scholarly reports since, for example, Yu et al. reported that the diameter of Polyacrylonitrile (PAN) aligned nanofibers continuously decreased as the speed of the rotating drum increased from 0 to 1200 r/min^49^. This could be partly due to the small variation of the speed in our experimental setup compared to that of Yu et al. More in depth analysis of the effect of the drum’s rotation speed has to be conducted in future studies to understand such potential influence.

Finally, different needle gauge numbers (20, 22, and 25) have resulted in PCL fibers of 1.4 μm, 1.6 μm, and 1.4 μm diameters, respectively. Considering the statistical difference between needle gauges 20 and 22 (p < 0.001), and 22 and 25 (p < 0.01), there is no significant difference between the examined groups; hence we presume the needle gauge to be of no significant impact on the obtained diameters of the fibers (Fig. 3D).

### Characterization of the aligned fibers by water contact angle (WCA) analysis

**Figure 4:**
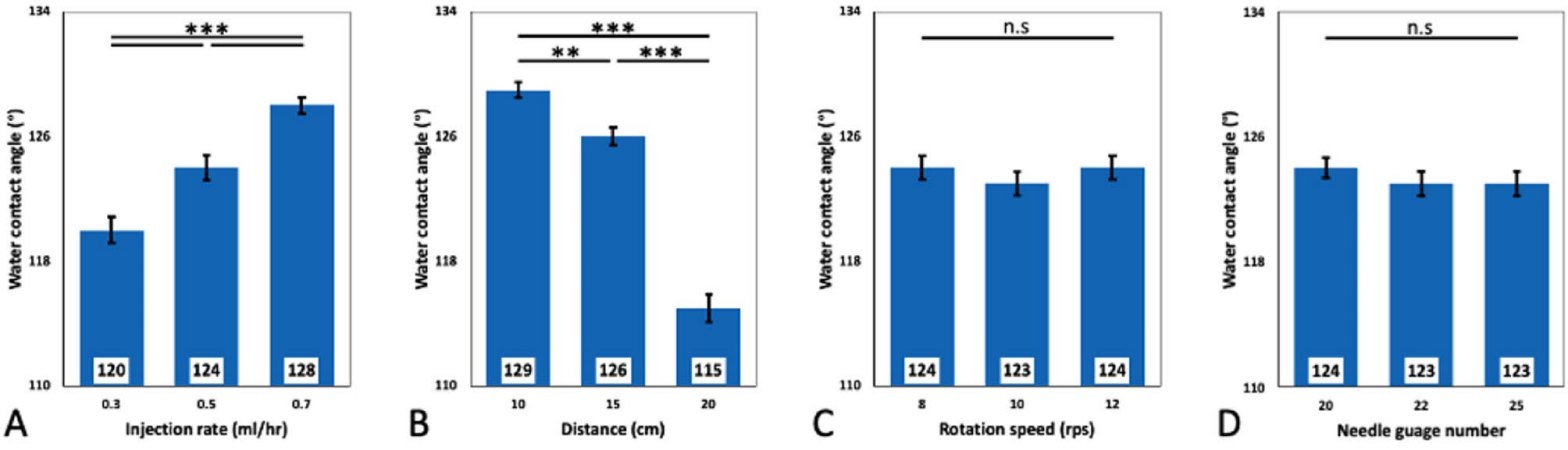
Measurement of water contact angle based on various influential electrospinning parameters. Bar graphs represent the effects of (**A**) Injection rate (ml/h), (**B**) needle-collector distance (cm), (**C**) drum rotation speed (rps), (**D**) needle gauge number on the diameter of the fiber. Statistical significance was determined using one-way ANOVA with Tukey posthoc analyses. **, ***, and ns indicates the significance level of p < 0.01, p < 0.001, and not significant, respectively. Data is shown in mean ± SEM (n = 405).

Several studies emphasized the importance of WCA of the electrospun fibers as the application of electrospun fibers is highly dependent on the hydrophobicity or hydrophilicity of the fiber’s surface^50,51^. This is an important characteristic of the fibers therefore, they can be tuned to favor different uses. The WCA measurements for the fabricated aligned PCL fibers were obtained over applied parameters. Figure 4A suggests that WCAs for injection rate of 0.3 ml/h, 0.5 ml/h, and 0.7 ml/h were 120°, 124°, and 128°, respectively, which shows an increase in injection rate (from 0.3 to 0.7 ml/h) significantly increased the WCA value (p < 0.001). This observation was very probable as more condensed fibers are produced due to higher injection rates. As the distance from the tip to the collector extended, lower WCAs were recorded while the fibers remained hydrophobic (Fig. 4B). Water contact angles of 129°, 126°, and 115° were resulted from distances of 10 cm, 15 cm, and 20 cm, respectively. In 2018, Ghobeira et al. reported that the WCA of aligned PCL fibers was decreased by shortening the distance between tip to the collector, which is contrary to our results^48^. While further experimental exploration can carefully determine such a correlation, from our result, we can argue that the long distance between the needle and collector creates elongated fibers; hence the gaps between the fibers could be relatively wider, which, in turn, would promote the water droplets to sink within such voids. This association can be clearly seen in our results presented in Figs. 3B and 4B. Our data also demonstrates that the altering rotation speed had no fundamental impact on the measured WCAs (no significant statistical difference) (Fig. 4C). Different rotation speeds of 8 rps, 10 rps, and 12 rps have generated WCAs of 124°, 123°, and 124°, respectively.

In contrast to our findings, Zhang et al. reported that increasing rotation speed (from 500 to 1000 and 2000 rpm) results in reduced WCA of PCL fibers due to the increased alignment^33^. There is a fundamental difference between the range of rps applied in the above-mentioned study as opposed to what we have selected as the experimental parameters that make us rely on the judgment of Zhang et al. as a solid observation. Lastly, no radical change in WCA was observed for different needle gauge numbers of 20, 22, and 25 (WCAs of 124°, 123°, and 123°), indicating that the size of the needle gauge had no influence on the recorded WCAs (Fig. 4D).

### Fast Fourier Transform (FFT), surface plot profile, and pixel intensity plots analyses

**Figure 5:**
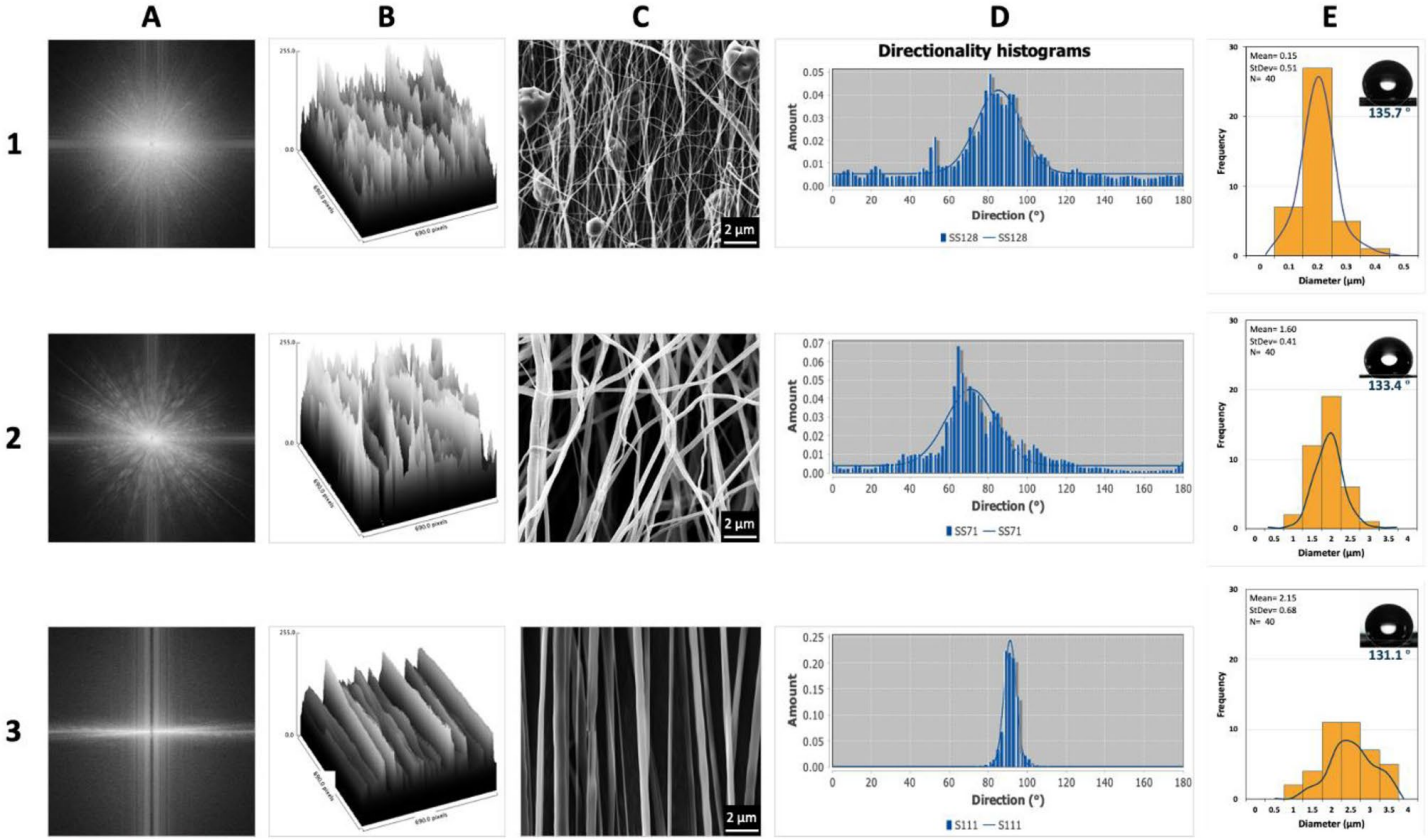
Alignment comparison of 3 PCL fabricated fibers from poor to medium and high alignment, respectively. (**A**) FTT output images; (**B**) Surface plot profile; (**C**) SEM image (×1000); (**D**) Pixel intensity plots based on the direction of the fibers; (**E**) Fiber diameter distribution (the inset shows water contact angle of the represented sample).

Figure 5 depicts 3 alignment scenarios for PCL electrospun fibers along with additional analyses. Column A in this image presents a 2-Dimensional Fast Fourier Transform (2D FFT) analysis which provides information on the fiber’s alignment. For comparison purposes, we gathered the analyses of a set of poorly aligned fibers (top row), the better-aligned fibers (middle row), and highly aligned fibers (bottom row). This shows the contrast between the results obtained as we tuned our experimental conditions for better alignment. Illustrated sample in the first row, which is a representative of poorly aligned PCL fibers, corresponds to a highly scattered FFT pattern of pixels (A1), while the pattern is less scattered in A2 and quite perpendicular in the case of A3.

The analysis presented in column B of Fig. 5 is the three-dimensional (3D) graph of pixels’ intensity that provides a topographic view of the fibers in each scenario. As the analysis moves from the top row (B1) to the middle (B2) and then to the last row (B3), more clear alignment is detected from this topographic analysis which agrees with the FFT and the SEM images presented in C column (C1, C2, and C3).

Another piece of information was gathered from the directionality graphs, which clearly goes hand in hand with the rest of the analyses (column D). As the fibers come to better alignment, the arbitrary orientation of the fibers comes to a narrower range (D1 and D2 to D3). This consistency can also be observed in the diameter range analysis of the fiber. More aligned fibers (E2 and E3) are, size-wise, closer to each other with a rather reliable consistency in the measurements (~ 1–3 μm), while the poorly aligned fibers (E1) are heavily beaded which, in turn, make the diameter range analysis somewhat questionable. WCA average for each scenario is in accordance with other analysis presented in Fig. 5. The poorly aligned fibers (E1) resemble a mesh structure with less gaps in between the fibers for the water to sink in (E1). As the fibers align, the in-between voids allow the water to permeate the fiber matrix slightly deeper hence lower WCA measurements.

**Figure 6:**
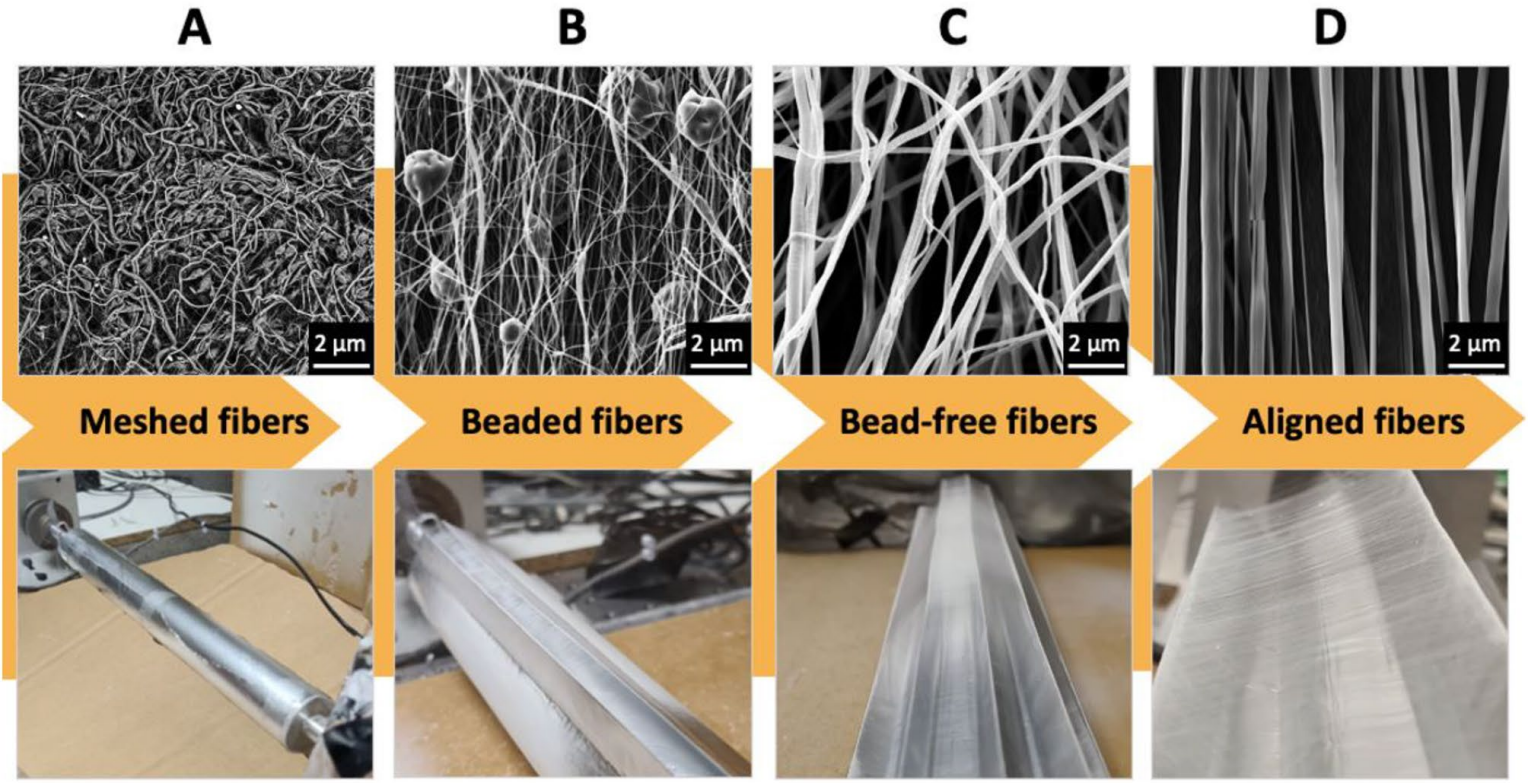
Development of origami folded aluminum foil mounted on a far-field electrospinning setup. The main objective was to eliminate the beads and fabricate highly aligned fibers. SEM images were added to represent a better visual comparison of alignment process.

The key contribution of this study is introducing a practical, low-cost, and easy-to-fabricate collector that highly influences the alignment of the fibers. In Fig. 6, we present our ideas' progression to the origami collector's development.

The regular rotating drum was used to produce the first set of fibers. According to a recent study^33^, aligned fibers could be produced as a result of higher rotation rates of the drum. After conducting several experiments at different speeds, the analyzed fibers presented meshed and beaded matrices that were far from favorable (Fig. 6A). We subsequently developed a two origami folded edges by using aluminum foil which was mounted on the rotating drum. This shape resulted in a better orientation of fabricated PCL fibers. However, the beads persisted (Fig. 6B). The beads were particularly undesirable as they impose poor control over the fibers quality. Since they are randomly scattered, they harshly affect other characterizations, including diameter range analysis and WCA measurements (as discussed). Some studies point out that the presence of beads results from the double-phased polymer solution or other possible contributing factors, including high surface tension, viscoelastic properties of the solution, and low charge density^52,53^. We attempted to remove the beads by fine-tuning the collectors. A tailored pentagon origami structure offered a much higher control over fiber deposition (Fig. 6C), while the resulted electrospun fibers were bead-free. Each edge was further tuned to have 1.5 cm height with 1 cm distance between the edges (Fig. 6D), meanwhile the radius of the drum remained the same (5 cm). This design facilitated the production of aligned fibers by far-field electrospinning without adding extra setups or equipment.

### Exploratory data analysis of the aligned fibers and feature selection

**Figure 7:**
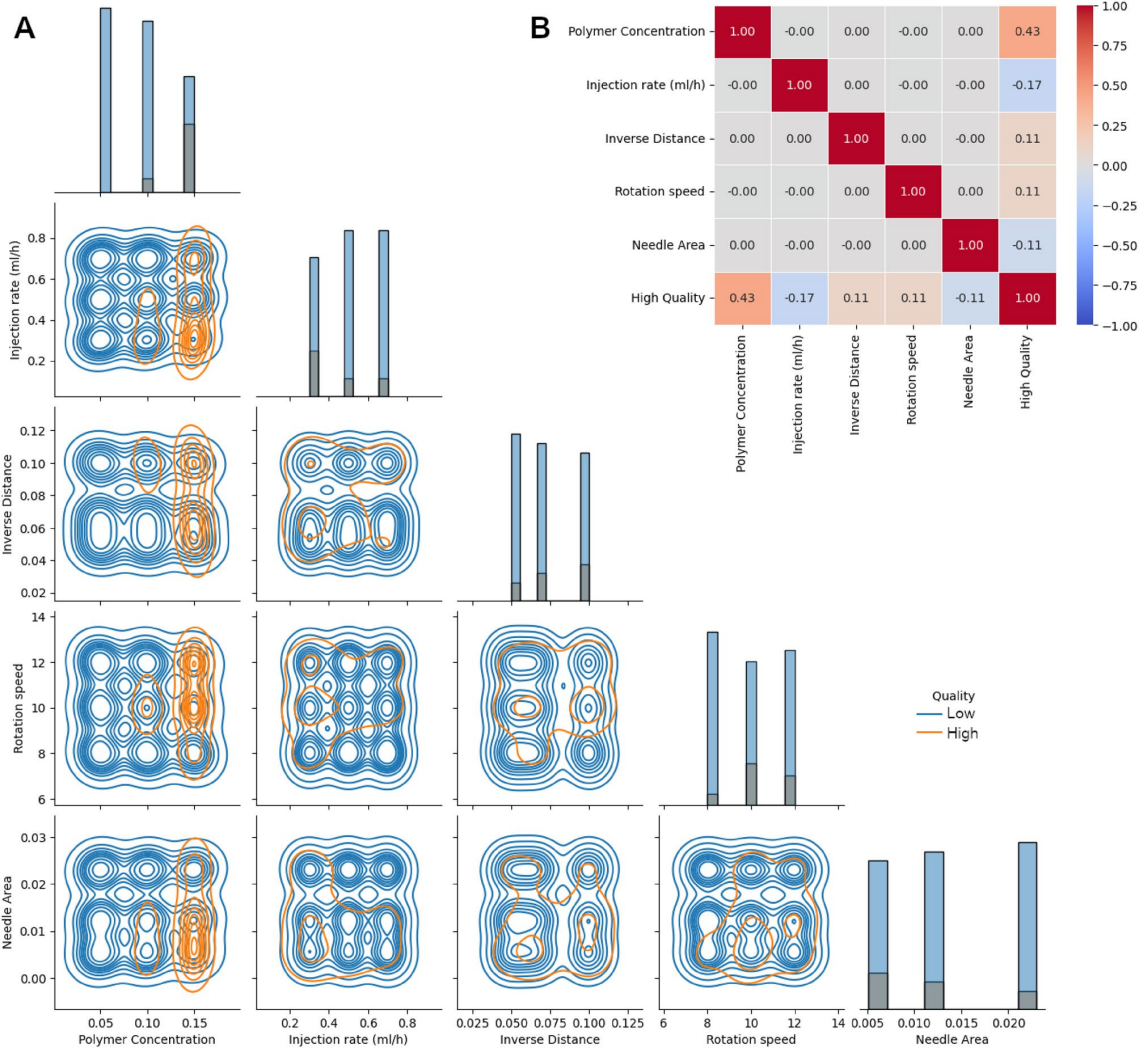
(A) Feature pair plot histograms, where blue and orange hues depict distributions for low- and high-quality samples, respectively. (**B**) Pearson correlation heatmap for experimental parameters.

According to the criteria described previously in the methodology, only 36 samples (14.8% of total) were obtained as high-quality fibers. Given the unbalanced nature of the quality class, it is imperative to prevent training biasing by employing two common-use strategies: Primarily, we utilize label stratification of validation, train, and test splits to ensure that these are balanced with consistent populations of good and bad quality labels, and subsequently, we balance the weights of the prediction class during LR and DT model fit to prevent biasing towards the majority label (i.e., low-quality). We observe that high quality is substantially correlated with polymer concentration, as evidenced in distribution histograms and correlation plots in Fig. 7. Moreover, we observe that other parameters show little significance in the outcome of the target variable. However, it is essential to note that Pearson correlation does not determine non-linear relations, which underestimates the effect of rotation speed towards the high-quality fibers, as seen in the non-linear nature of Fig. 7A,B.

Choosing the most meaningful features that provide a model to explain the experimental data is important. By assuming linear relation of parameters with target class, after polymer concentration, Rotation Speed and Injection Rate come as the most significant paramters, meanwhile, Needle Gauge and Distance are seemingly the least important ones, which is in agreement with our observations from ANOVA testing. We choose RFECV as a systematic method that determines which is the most significant feature subset based on the performance of the chosen classifiers rather than simply relying on collinearity analysis, which underestimates non-linear effects on the target class.

### Logistic regression and decision tree algorithms

**Figure 8:**
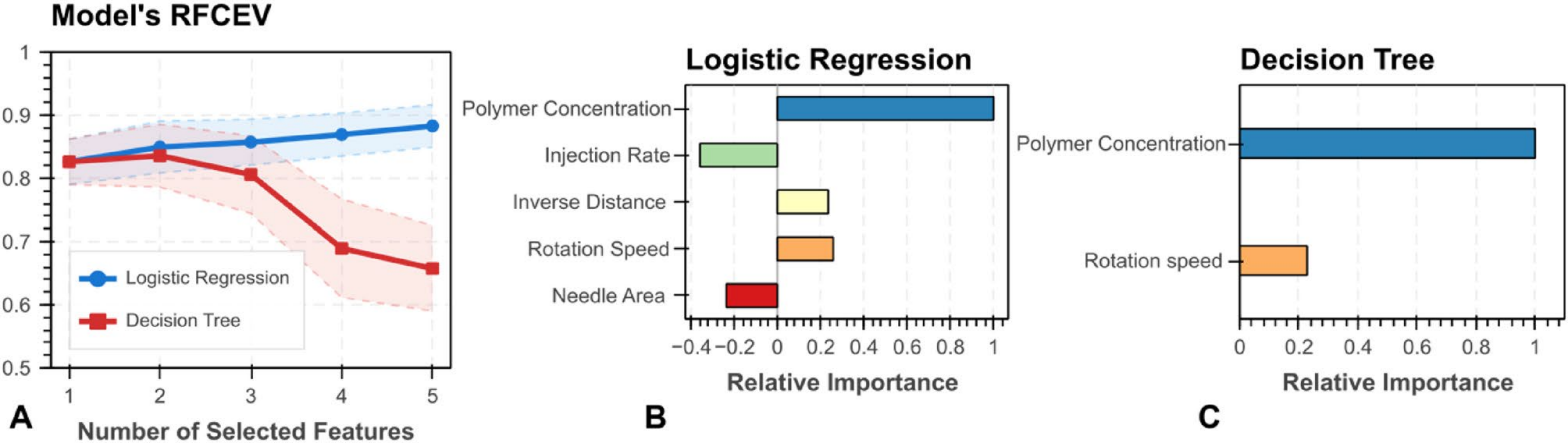
(**A**) Recursive feature elimination with cross-validation of Logistic Regression and Decision Tree estimators. Markers depict the mean ROC AUC score and bands the ± 1 standard deviation from 100 iterations of 3-folded cross validation. Feature importance as obtained from (**B**) Logistic Regression and considering and (**C**) Decision Tree estimators.

When using LR as RFECV estimator, it leads to an increasing monotonic behavior of ROC AUC score when changing the number of considered features. In DT case, the score plateaus at two features, corresponding to Polymer Concentration and Rotation Speed as seen in Fig. 8A, while the Injection Rate ranks as the closest third most significant feature (not shown). One must bear in mind that Injection Rate is an important feature that determines fiber properties, including diameter; however, our labeling scheme was rather restricted to a simple, binary ruleset that favours homogeinity and disregards finer phyisical details (e.g., fiber size, array periodicity, water contact angle, etc.) hence drum rotation speed comes as a more significant parameter according to our quality-determining structure. Since LR RFECV does not display a maxima score, we incline to employ DT RFECV feature suggestions as a way to prevent overfitting (see recommended features in Fig. 8B,C). Hence, we chose Polymer Concentration and Rotation Speed as the significant features that determine fiber quality within our experimental constraints.

**Figure 9:**
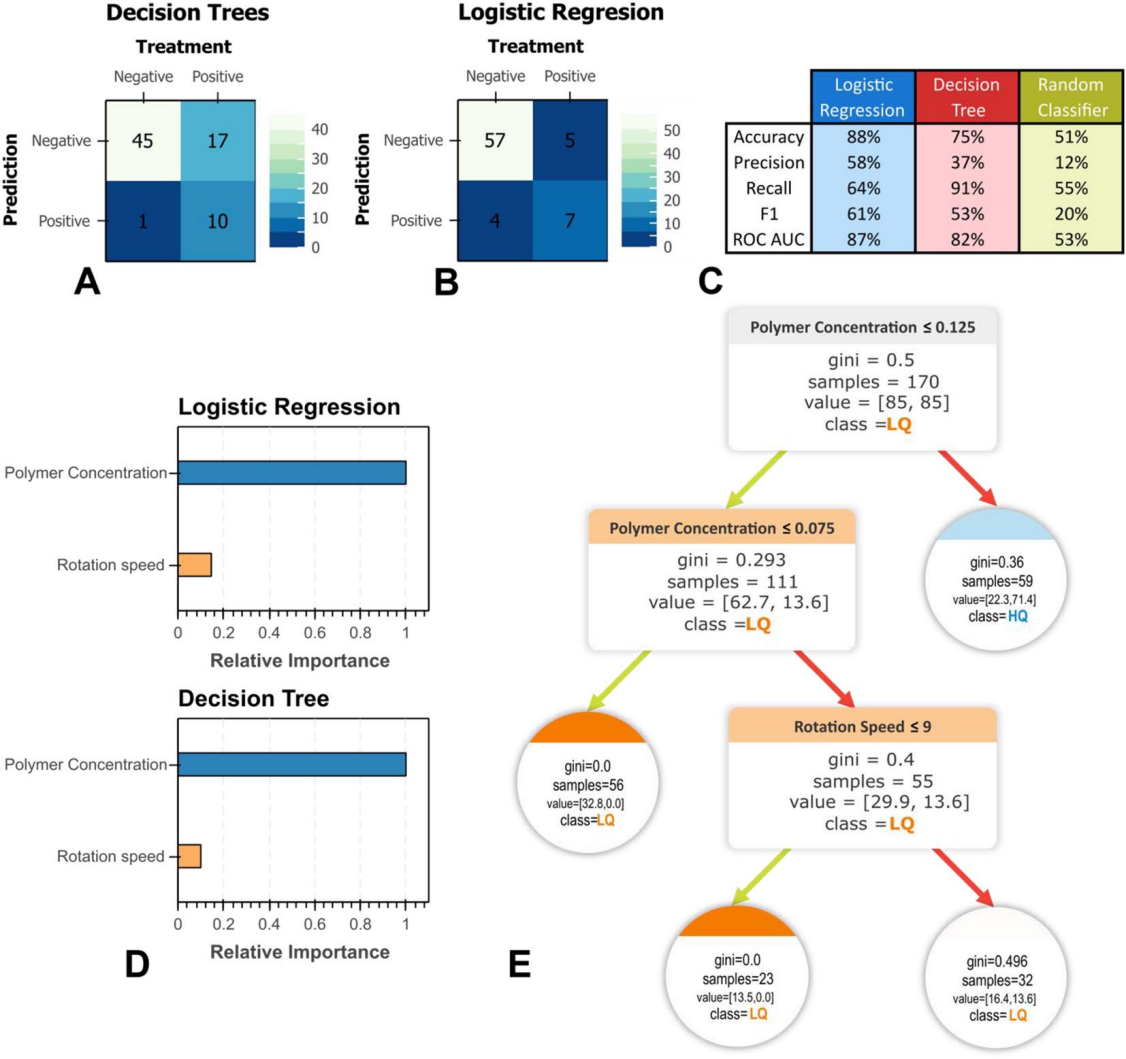
Confusion Matrixes for (**A**) Logistic Regression and (**B**) Decision Tree models and their (**C**) respective classification report for prediction with the validation set, including a randomized dummy classifier as a comparison baseline. (**D**) Relative feature importance plots and (**E**) decision tree plot.

After running the hyperparameter optimization procedure with the train/test split (as described in methodology and briefly seen in Fig. 7), we benchmark the LR and DT models' performance with the unseen validation split and compare them with a random dummy classifier as seen in Fig. 8C. When comparing ML models against the dummy baseline, it overall scores better, hinting that the complex models can learn and generalize the experimental data. As seen in confusion matrix plots (Fig. 9A,B) and classification report (Fig. 9C), the LR model predicts high-quality target with an accuracy of 88% and precision of 58%, outperforming DT scores of 75% and 37%, respectively. However, this comes with a tradeoff, as DT exhaustively covers more true positive cases than LR, thus performing with a higher recall score (91%) when compared to LR (64%).

To explain the performance difference between the two models, we can compare the importance of the features between LR and DT models by homogenizing the logistic regression coefficients with DT’s feature importance, which is simply done by normalization with the value of the biggest coefficient (i.e., Polymer Concentration) as seen in Fig. 9D. As previously evidenced by correlation data (see Fig. 8B), Polymer Concentration is the most important factor in establishing good quality aligned electrospun fibers. We can appreciate from Fig. 9D that drum rotation speed plays a slightly significant role in the determination of quality as well. Moreover, this feature is more impactful in LR than in DT (14.6% and 9.6%, respectively), partly explaining the difference in the predictive behavior of both models.

While the expectation of a DT model is to perform better in situations where non-linearity is present, it is evident that the rotation speed feature does not play a discriminatory role, as seen in Fig. 9E, thus explaining the exhaustive nature of this model when comparing against the more precise LR model. In contrast, LR takes into account both parameters to predict mat quality. The DT model offers complemental insights regarding which circumstances did not ensure good quality alignment. The first was polymer concentration is low (less than 0.075%), and the second, when polymer concentration is at the “*gray zone”* or threshold (around 0.1%), while the alignment quality is relatively poor if the drum rotation speed is not fast enough (i.e., larger than 9 rps). One must consider that, due to our experimental constraints, the DT model is limited in shedding light on what conditions are required to lead to a high alignment at this regime (as evidenced by the equally balanced label frequencies, as seen as a Gini leaf node score of 0.493 in Fig. 9E), or even how better predict good quality at our highest experimental concentration (0.125%). According to the magnitude of LR coefficients, we should anticipate that exploring additional concentration points beyond our experimental limits can help us find a more generalizable DT model that connects the conditions and leads to better alignment at high concentrations of the polymer^54^. In the light of connecting ML with material science, we identified the most significant factors relevant to yield acceptable alignment with our proposed drum setup, which opens the way for future exploration towards a better understanding of good quality deposition of electrospun mats.

## Conclusion

In this work, we present a new method for the fabrication of highly aligned fibers without modifying the conventional far-field electrospinning setup. This technique relies upon a simple and cost-effective origami-based design that mounts on the top of the rotating drum and allows a high degree of control over the quality of the fibers without the need for complex setups or multi-phased fabrication. We have designed 243 sets of experiments through variations of the experimental parameters and fabricated a range of fibers from PCL. These fibers were carefully analyzed by SEM, fiber diameter distribution, WCA, FFT, surface plot profile, and pixel intensity plots. Furthermore, we employed machine learning models to identify the influential parameters that were instrumental to high-quality aligned fiber fabrication. From the 243 rounds of experiments, 10 conditions were selected as the ideal aligned fibers. Our thorough assessments suggest that polymer concentration plays the most significant role in the quality of the aligned fibers followed by the rotation speed of the collecting drum. This study offers multiple points of novelty: (i) no previously reported methodology competes with our proposed technique in the sense of simplicity and cost-effectiveness; (ii) the disclosed method is the first to leave the conventional far-field electrospinning setup intact and without any modification which is a major advantage in laboratories where multiple projects are conducted on same machinery; (iii) the proposed origami-based collector yields fibers of fine alignment and offers a close control over fibers’ parameters which opens various windows of opportunity for fibers’ application; and (iv) the current work brings two powerful areas of material science and machine learning together with specific insights on the role of applied algorithms in predicting high-quality fibers alignment.

## Supplementary Information

Supplementary Information.

## Data Availability

The datasets used and/or analysed during the current study available from the corresponding author on reasonable request.

## Abbreviations

2D FFT: 2-Dimensional Fast Fourier Transform
3D: 3-Dimensional
BPUR: Biodegradable polyurethane
CNTs: Carbon nanotubes
DMF: N,N Dimethylformamide
DT: Decision trees
FFT: Fast Fourier Transform
FN: False negative
FP: False positive
HQ: High quality
LQ: Low quality
LR: Logistic regression
ML: Machine learning
PAN: Polyacrylonitrile
PCL: Poly(ɛ-caprolactone)
PEM: Parallel electrode method
PEO: Poly(ethylene oxide)
PMMA: Poly(methyl methacrylate)
PS: Polystyrene
RFECV: Recursive feature elimination with cross-validation
ROC AUC: Receiver operating characteristic curve
SEM: Scanning electron microscope
THF: Tetrahydrofuran
TN: True negative
TP: True positive
WCA: Water contact angle
