# Supplementary material for: An origami-based technique for simple, effective and inexpensive fabrication of highly aligned far-field electrospun fibers
Source: Sci Rep. 2023 May 1;13:7083. doi: 10.1038/s41598-023-34015-z (PMC10151330; doi:10.1038/s41598-023-34015-z)
Supplement: Supplementary file 1 — Supplementary Information. [file 41598_2023_34015_MOESM1_ESM.docx]

**An origami-based technique for simple, effective and inexpensive fabrication of highly aligned far-field electrospun fibers**

Hamed Hosseinain^1^, Martin Jimenez-Moreno^1^, Mazhar Sher^2^, Aida Rodriguez-Garcia^1,3^, Sergio O. Martinez-Chapa^1^, Samira Hosseini^1,4,*^

^1^School of Engineering and Sciences, Tecnologico de Monterrey, Monterrey, NL, 64849, Mexico

^2^Department of Agricultural and Biosystems Engineering, South Dakota State University, SD 57007, United States.

^3^Universidad Autonoma de Nuevo Leon, Facultad de Ciencias Bioloogicas, Instituto de Biotecnología, Ave. Pedro de Alba S/N, Ciudad Universitaria, San Nicolaas de los Garza, NL, 66455, Mexico.

^4^Writing Lab, Institute for the Future of Education, Tecnologico de Monterrey, Monterrey, NL, 64849, Mexico

**Supplementary Section**


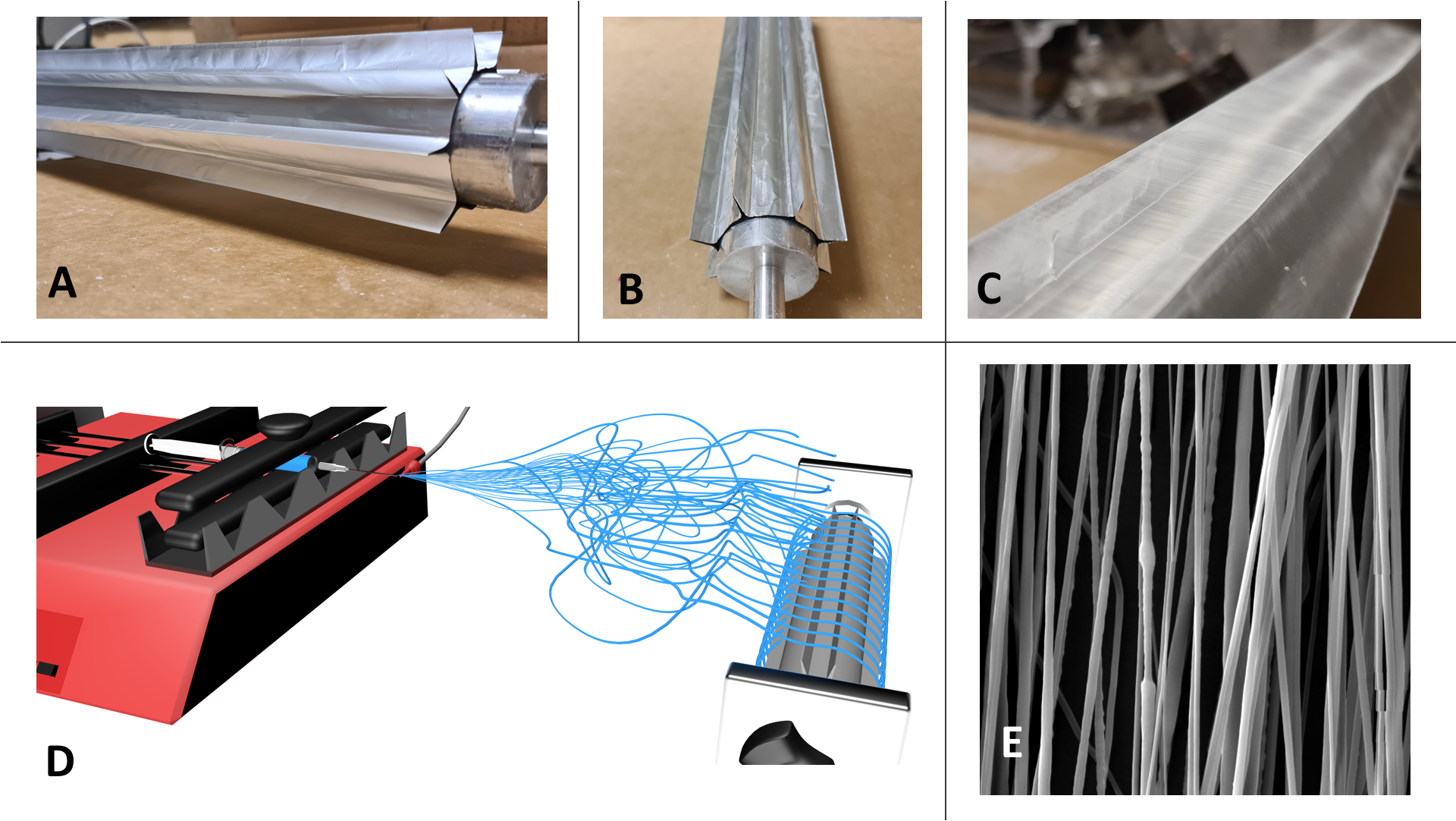


**Supplementary Figure 1.** The details of the fabrication setup including: A and B) the origami collector; C) the zoomed in view of the fibers; D) the schematic representation of the setup; and E) the SEM image of the aligned fibers deposited in between the edges of the origami collector

**Supplementary Table 1.** The main influential parameters in aligned fiber fabrication: Polymer concentration (5%, 10% and 15%), the injection rate (0.3, 0.5, and 0.7 ml/h), and the distance between the tip and the collector (10, 15, and 20 cm). Note, that 5% polymer solution did not result in fibers and instead powder-like constructs were deposited on the collector.

| **Injection rate (ml/h)** | **Concentration 5%** | | | **Concentration 10%** | | | **Concentration 15%** | | |
| --- | --- | --- | --- | --- | --- | --- | --- | --- | --- |
| 0.7 | 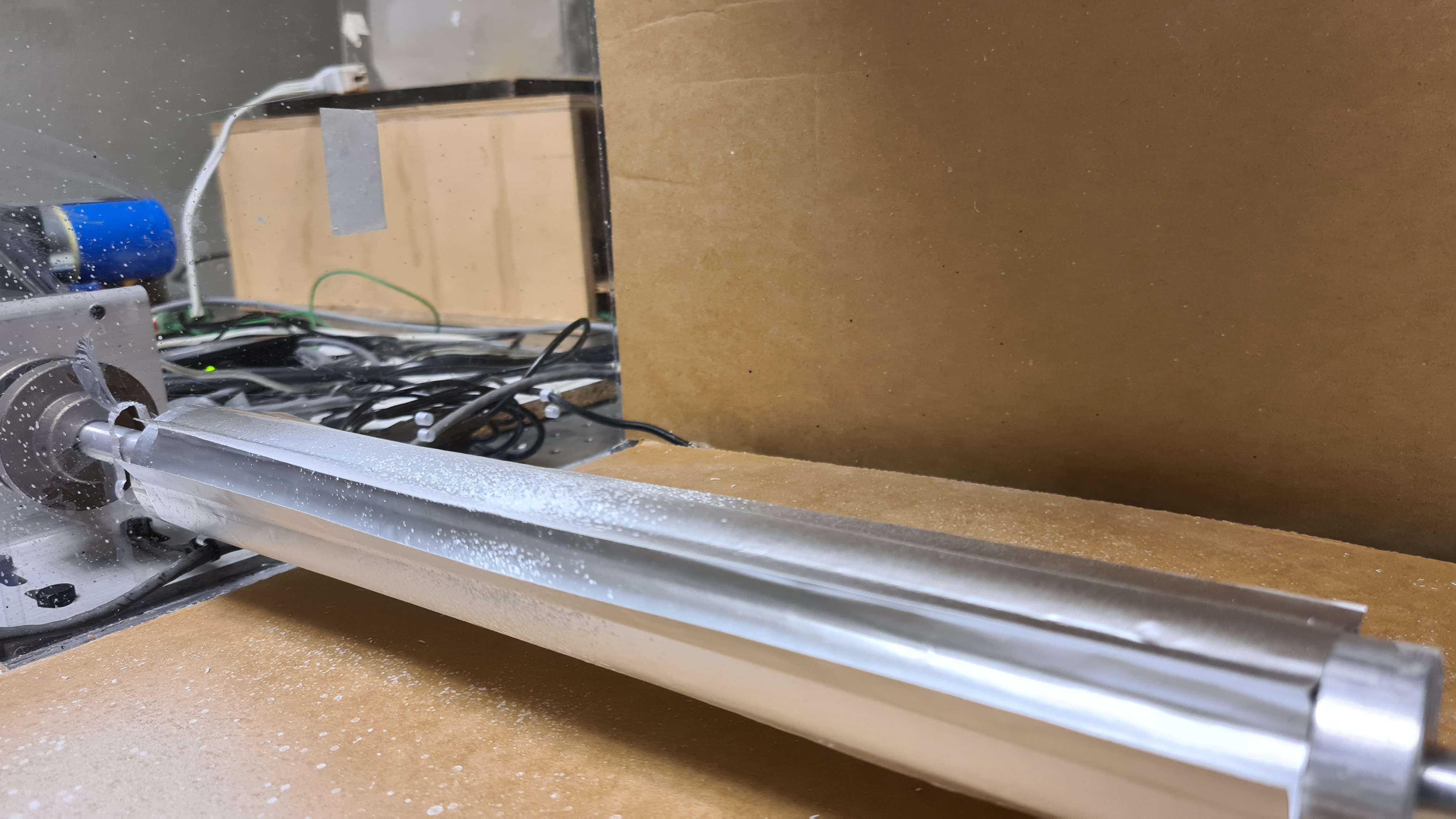 | 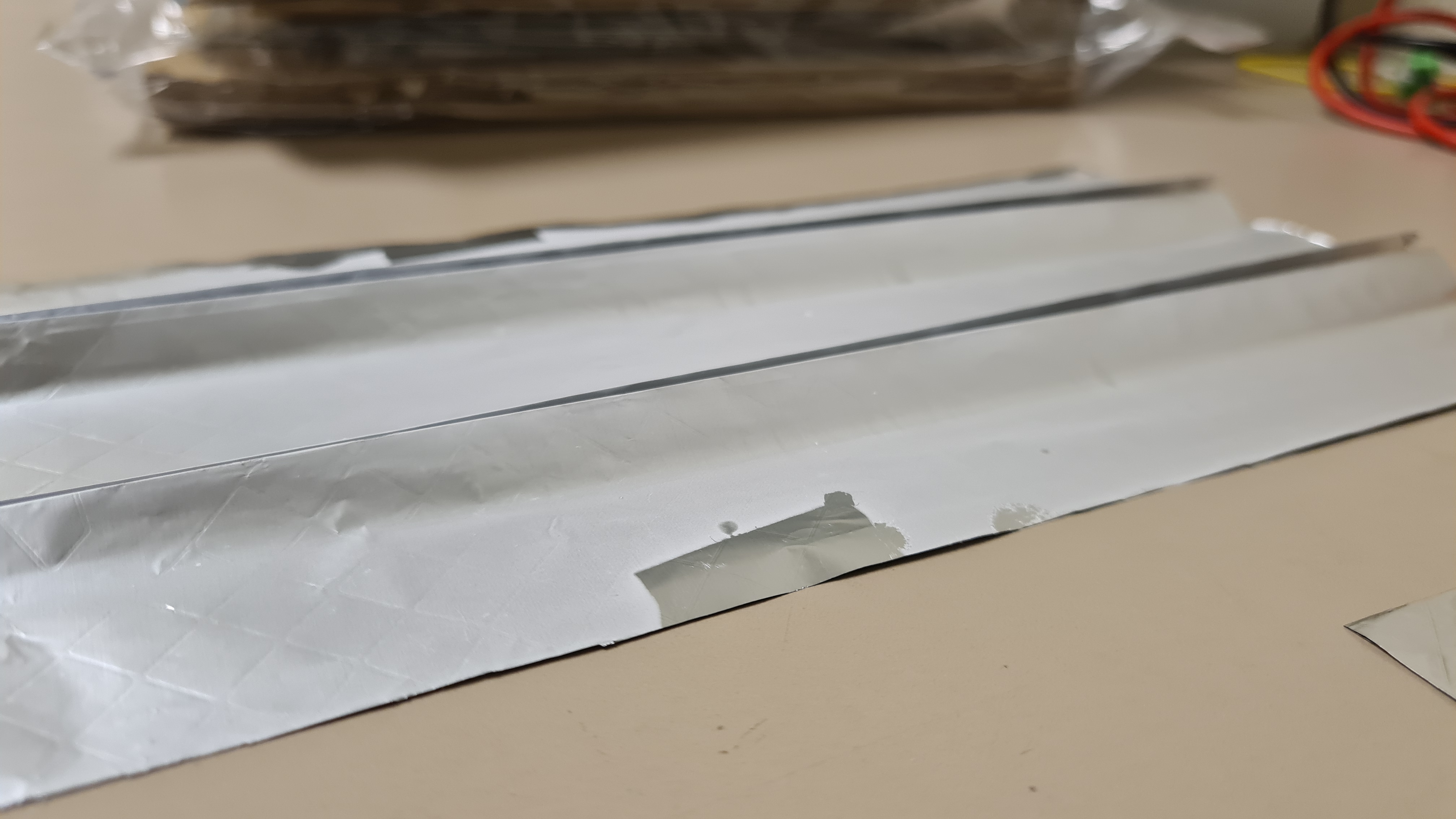 | 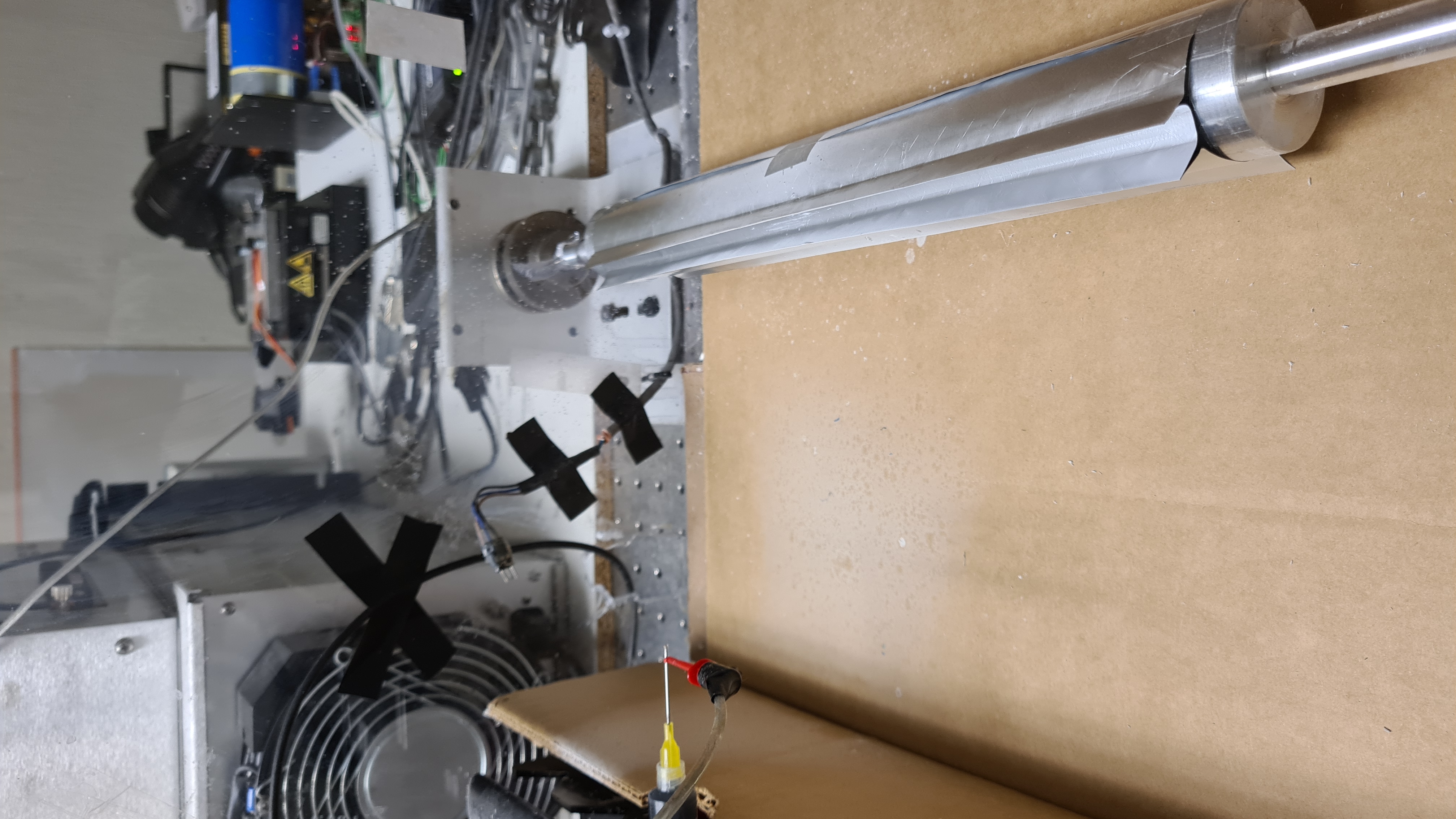 |  |  |  |  |  |  |
| 0.5 | 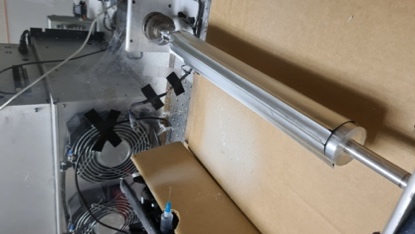 | 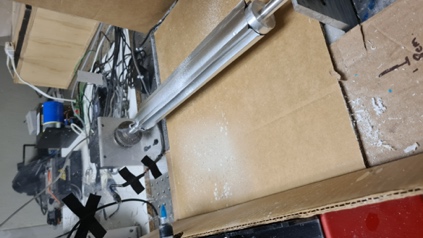 | 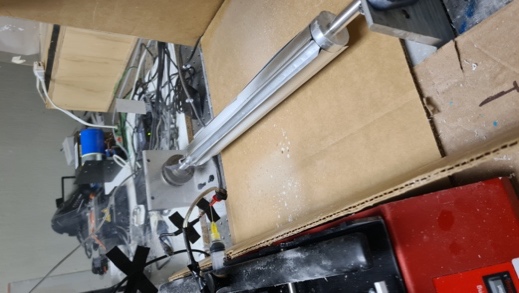 | 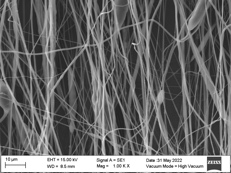 | 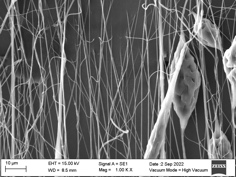 | 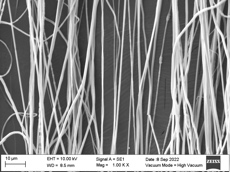 | 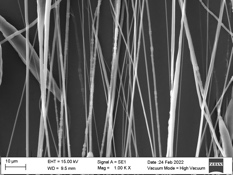 | 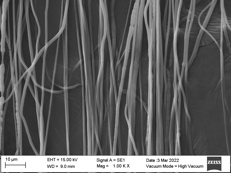 |  |
| 0.3 | 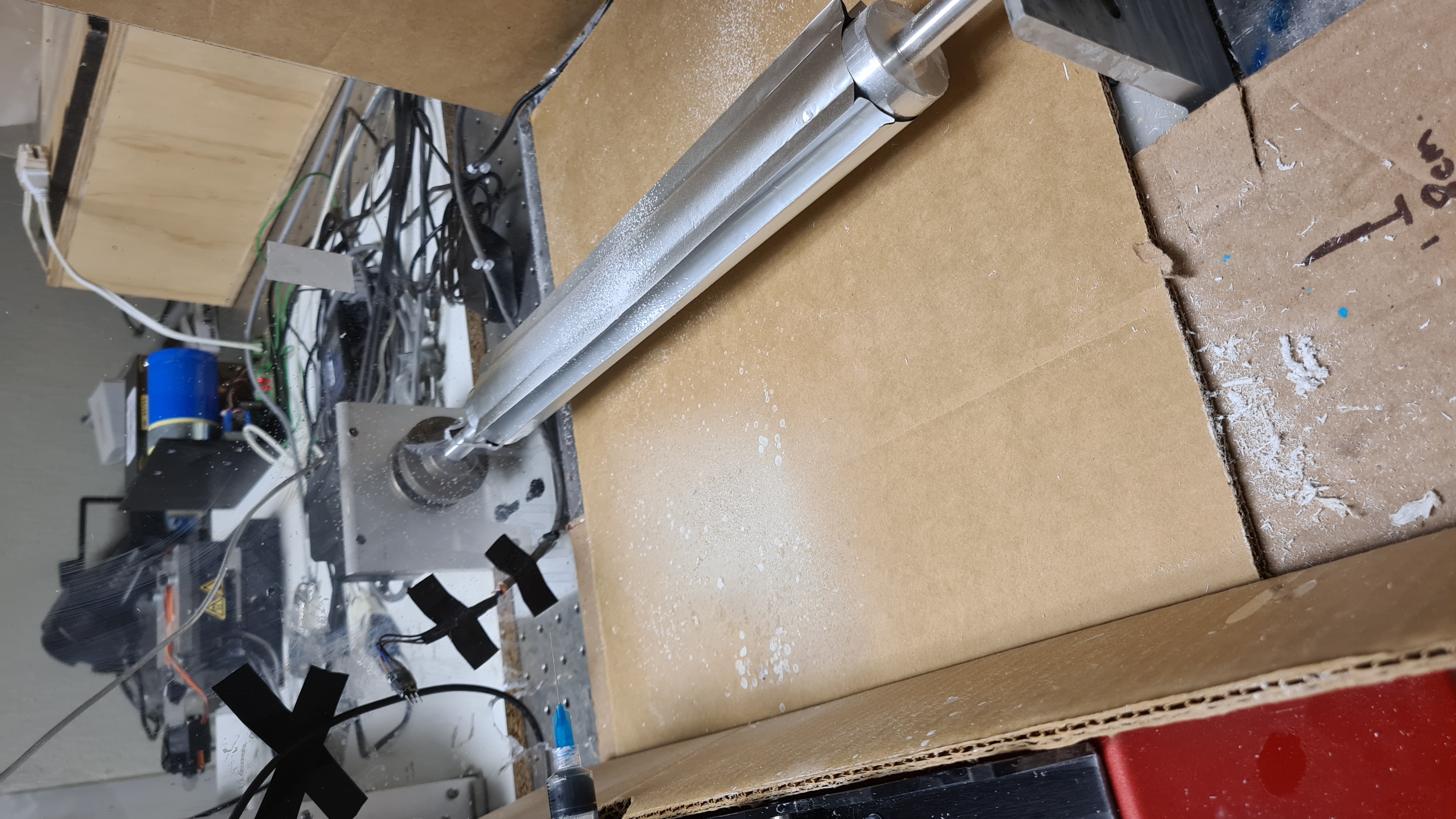 | 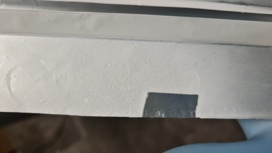 | 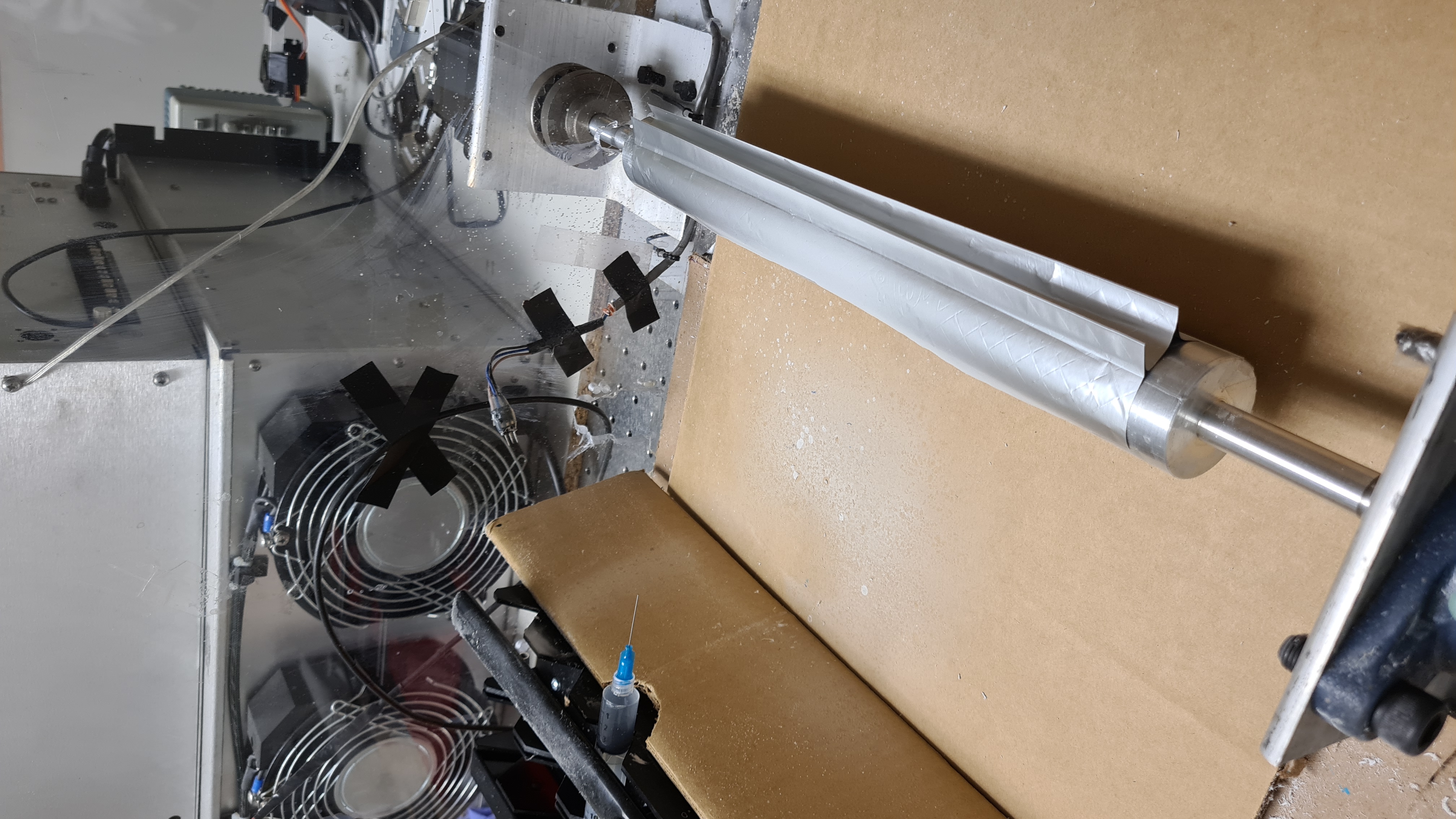 |  |  |  |  |  |  |
| **Distance (cm)** | 10 | 15 | 20 | 10 | 15 | 20 | 10 | 15 | 20 |
